# Supplementary material for: Extracellular Vesicle-Mediated Purinergic Signaling Contributes to Host Microenvironment Plasticity and Metastasis in Triple Negative Breast Cancer
Source: Int J Mol Sci. 2021 Jan 9;22(2):597. doi: 10.3390/ijms22020597 (PMC7827112; doi:10.3390/ijms22020597)
Supplement: Supplementary file 1 [file ijms-22-00597-s001.pdf]

Supplementary Tables and Figures with Legends

Supplementary Table S1. Top 20 up-regulated lung proteins after MDA-MB-231 EV treatment. Mice were treated for three-weeks with MDA-MB-231 EVs or PBS vehicle and TMT-labeled lungs were submitted for mass spectrometry analysis. Significantly enriched proteins in MDA-MB-231 EV-treated lungs with raw p-values less than 0.05 were ranked by log2 fold-change.

Protein Name	Gene Name	log2 FC	P Value
Alpha-1-antitrypsin 1-5	Serpina1e	1.00	0.0404
Interferon-inducible GTPase 1	Iigp1	0.89	0.0110
Glycoprotein Ib, beta polypeptide	Gp1bb	0.83	0.0388
Solute carrier family 2, facilitated glucose transporter member 3	Slc2a3	0.77	0.0164
Interferon-induced protein 44-like	Ifi44l	0.77	0.0021
FYN-binding protein	Fyb	0.76	0.0228
Tubulin beta-1 chain	Tubb1	0.72	0.0091
Integrin alpha-Iib	Itga2b	0.72	0.0083
Integrin beta-3	Itgb3	0.71	0.0041
C-type lectin domain family 1 member B	Clec1b	0.70	0.0035
Lymphocyte cytosolic protein 2	Lcp2	0.69	0.0027
Thrombospondin 1	Thbs1	0.68	0.0048
Bridging integrator 2	Bin2	0.66	0.0067
Metalloreductase STEAP4	Steap4	0.64	0.0028
Platelet glycoprotein IX	Gp9	0.61	0.0344
Fermitin family homolog 3	Fermt3	0.58	0.0029
Phospholipase D4	Pld4	0.58	0.0003
DNA replication licensing factor MCM3	Mcm3	0.57	0.0226
Multimerin-1	Mmrn1	0.56	0.0361
DNA replication licensing factor MCM4	Mcm4	0.55	0.0238

Supplementary Table S2. Top 20 down-regulated lung proteins after MDA-MB-231 EV treatment. Mice were treated for three-weeks with MDA-MB-231 EVs or PBS vehicle and TMT-labeled lungs were submitted for mass spectrometry analysis. Significantly down-regulated proteins in MDA-MB-231 EV-treated lungs with raw p-values less than 0.05 were ranked by log2 fold-change.

Protein Name	Gene Name	log2 FC	P Value
Migration and invasion enhancer 1	Mien1	-0.34	0.0047
Stonin-1	Ston1	-0.37	0.0254
Polymeric immunoglobulin receptor	Pigr	-0.32	0.0145
PTB domain-containing engulfment adapter protein 1	Gulp1	-0.29	0.0006
Voltage-dependent calcium channel subunit alpha-2/delta-1	Cacna2d1	-0.29	0.0139
Carboxylic ester hydrolase	Ces2g	-0.26	0.0021
UPF0585 protein C16orf13 homolog		-0.25	0.0193
CD82 antigen	Cd82	-0.25	0.0408
Hepatocyte growth factor activator	Hgfac	-0.24	0.0357
Alkaline phosphatase, tissue-nonspecific isozyme	Alpl	-0.24	0.0573
Tumor-associated calcium signal transducer 2	Tacstd2	-0.23	0.0040
CDP-diacylglycerol--inositol 3-phosphatidyltransferase	Cdipt	-0.23	0.0085
Annexin A9	Anxa9	-0.23	0.0225
Angiotensin-converting enzyme	Ace	-0.23	0.0595
Septin-5	Sept5	-0.22	0.0164
Heat shock protein beta-8	Hspb8	-0.22	0.0626
Transforming growth factor beta receptor type 3	Tgfbr3	-0.22	0.0008
Serine/threonine-protein phosphatase 2A	Ppp2r5c	-0.22	0.0259
Epoxide hydrolase 1	Ephx1	-0.21	0.0046
Claudin-5	Cldn5	-0.21	0.0031

Supplementary Table S3. Top 20 proteins with greatest fold enrichment in MDA-MB-231 EVs. Significantly enriched proteins in MDA-MB-231 EVs with adjusted p-values less than 0.01 were ranked by log2 fold-change.

Protein Name	Gene Name	log2 FC	P Value
Thrombospondin-1	THBS1	7.1	9E-05
Complement C4-A	C4A	5.4	9E-05
Insulin-like growth factor-binding protein 4	IGFBP4	5.2	9E-05
Protein CYR61	CYR61	4.9	9E-05
Tissue-type plasminogen activator	PLAT	4.6	9E-05
Connective tissue growth factor	CTGF	4.2	9E-05
Pentraxin-related protein PTX3	PTX3	3.9	9E-05
Sulfhydryl oxidase 1	QSOX1	3.8	9E-05
Transforming growth factor beta 1	TGFB1	3.8	9E-05
Neuropilin-1	NRP1	3.8	9E-05
Eukaryotic translation initiation factor 4B	EIF4B	3.7	9E-05
Integrin beta 1	ITGB1	3.7	9E-05
Filamin-C	FLNC	3.7	9E-05
Threonine-tRNA ligase	TARS	3.6	9E-05
Properdin	CFP	3.5	9E-05
Nucleosome assembly protein 1-like 1	NAP1L1	3.5	3E-04
Protein-lysine 6-oxidase	LOX	3.3	1E-04
Insulin-like growth factor-binding protein 7	IGFBP7	3.3	9E-05
Dickkopf-related protein 1	DKK1	3.3	8E-04
Protein-glutamine gamma-glutamyltransferase 2	TGM2	3.2	3E-04

Supplementary Table S4. Top 20 proteins with greatest fold enrichment in MCF-12F EVs. Significantly enriched proteins in MCF-12F EVs with adjusted p-values less than 0.01 were ranked by log2 fold-change.

Protein Name	Gene Name	log2 FC	P Value
Laminin subunit beta-3	LAMB3	6.5	9E-05
Laminin subunit alpha-3	LAMA3	6.4	9E-05
Laminin subunit gamma-2	LAMC2	6.4	9E-05
Fibulin-1	FBLN1	6.3	9E-05
Sushi, von Willebrand factor type A, EGF and pentraxin domain-containing protein1	SVEP1	6.0	9E-05
Clusterin	CLU	5.6	9E-05
Complement C1s subcomponent	C1S	5.6	9E-05
Mesothelin	MSLN	5.2	9E-05
Bone morphogenetic protein 1	BMP1	5.1	9E-05
Latent-transforming growth factor beta-binding protein 1	LTBP4	4.8	9E-05
Syntenin-1	SDCBP	4.8	9E-05
ADATS-like protein 4	ADAMTSL4	4.8	9E-05
Secreted frizzled-related protein 1	SFRP1	4.7	9E-05
Keratin, type II cytoskeletal 17	KRT17	4.6	9E-05
Aminopeptidase	ANPEP	4.6	9E-05
Procollagen-lysine, 2-oxoglutarate 5-dioxygenase 1	PLOD1	4.6	9E-05
Heat shock protein beta-1	HSPB1	4.6	9E-05
Glia-derived nexin	SERPINE2	4.5	9E-05
CD109 antigen	CD109	4.3	9E-05
Tsukushin	TSKU	4.2	5E-04

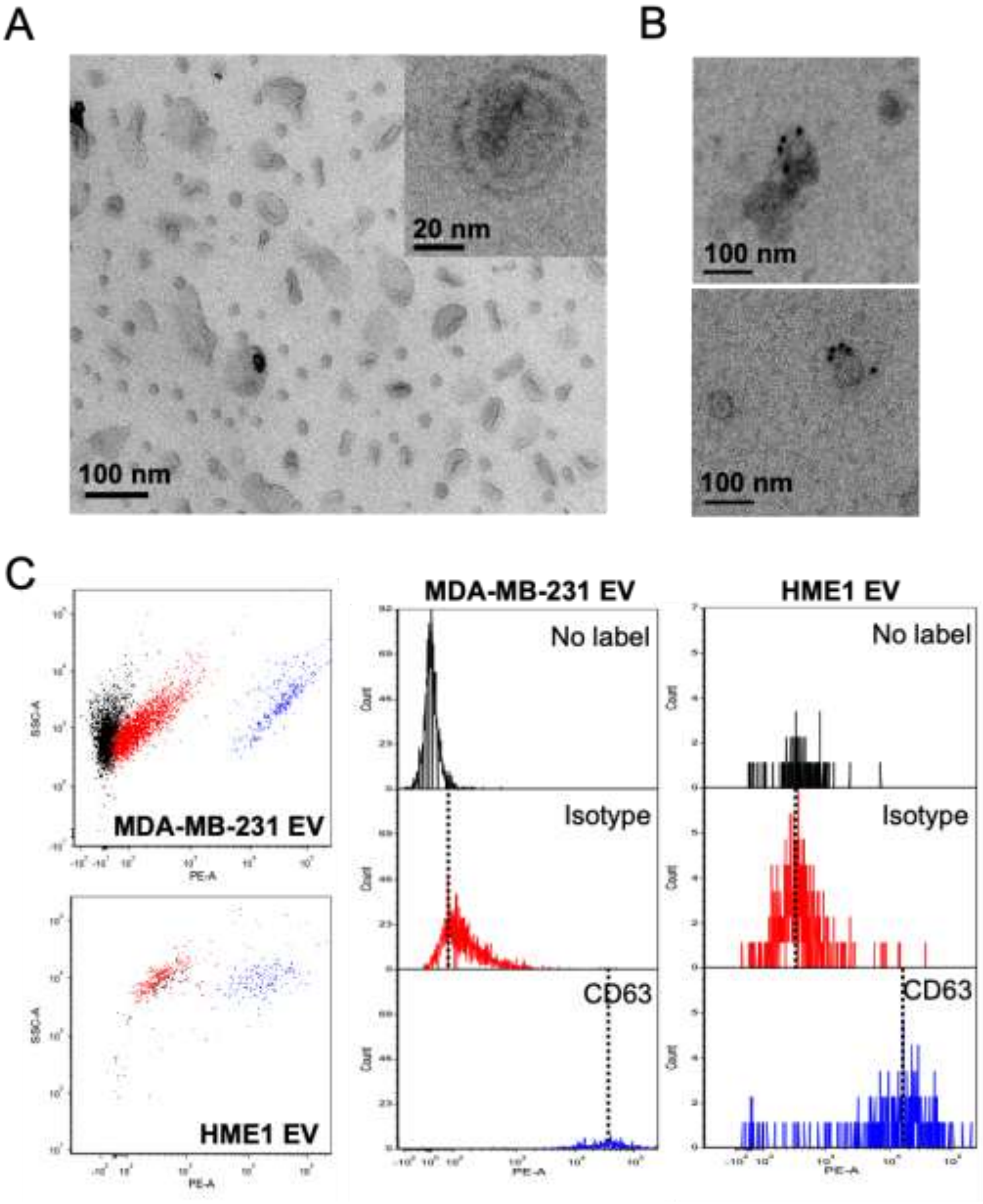

Supplementary Figure S1. Characterization of ExoQuick-TC-isolated HME1 and MDA-MB-231 EVs by TEM, CLSM, and flow cytometry. *(A)* TEM images of EVs isolated from MDA-MB-231 cells. Scale 20 and 100 nm. *(B)* MDA-MB-231 and HME1 EVs immuno-gold labeled for tetraspanins, respectively. Scale 100 nm. *(C)* Flow cytometry histogram plots comparing CD63 expression in MDA-MB-231 and HME1 EV populations.

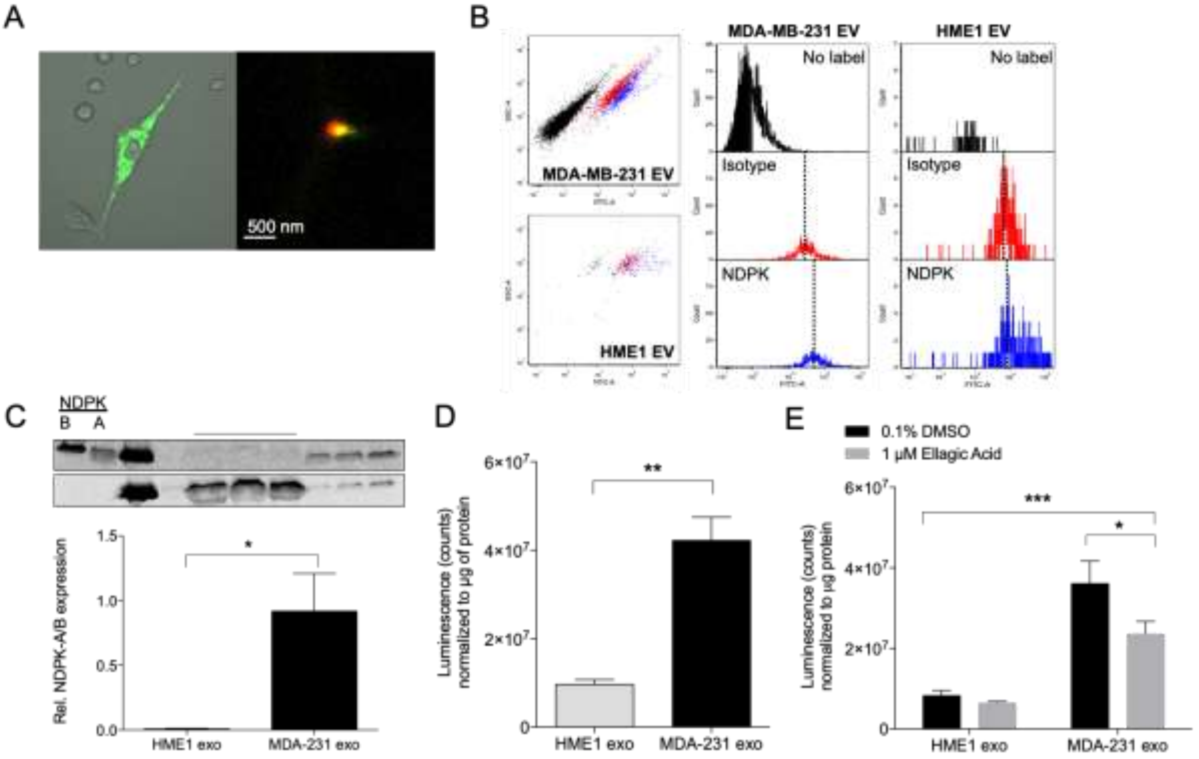

Supplementary Figure S2. MDA-MB-231 EVs isolated by ExoQuick-TC are enriched in NDPK-A/B expression and transphosphorylase activity. *(A)* Super-resolution image of a single MDA-MB-231-CD63-GFP expressing cell (left) and a representative MDA-MB-231 EV (right) immuno-stained for NDPK-B (red) expression. Scale 500 nm. *(B)* Flow cytometry analysis of MDA-MB-231 and HME1 EV populations immuno-labeled for NDPK-A expression. *(C)* Western blot analysis of lysed MDA-MB-231 and HME1 EVs and relative quantitation of NDPK-A/B expression. Recombinant NDPK-A, NDPK-B, and MDA-MB-231 cell lysate (CL) are also shown for comparison. n = 3. Mean ± S.D. *$p < 0.05$ by two-tailed Student's t-test. *(D)* Measurement of NDPK activity in MDA-MB-231 and HME1 EVs using an ATP transphosphorylase activity assay. n = 3. Mean ± S.D. *$p < 0.05$ by two-tailed Student's t-test. *(E)* Measurement of transphosphorylase activity in MDA-MB-231 and HME1 EVs treated with ellagic acid, an inhibitor of NDPK, or with DMSO vehicle. n = 3. Mean ± S.D. *$p < 0.05$, **$p < 0.01$, ***$p < 0.001$ by two-way ANOVA.

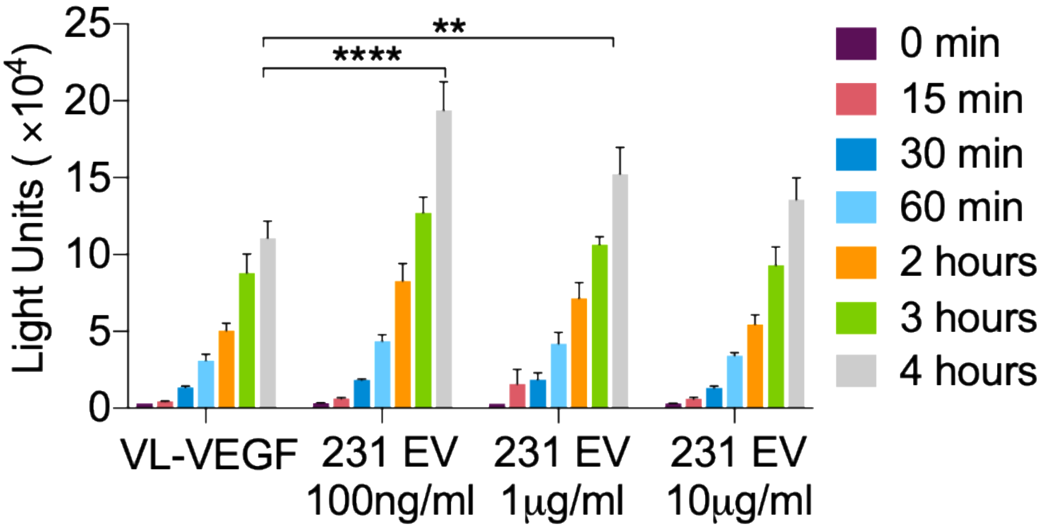

Supplementary Figure S3. The effect of varying MDA-MB-231 EV concentrations on endothelial monolayer permeabilization to FITC-dextran. HUVEC monolayers seeded in Matrigel-coated tranwell chambers were treated with MDA-MB-231 EVs in complete growth medium (VL-VEGF). Intensity of FITC-dextran permeabilization was measured at indicated time points as a measure of enhanced permeability. n = 3. Mean ± S.E.M. **p < 0.01 and ***p < 0.001 by one-way ANOVA and Tukey post-test.

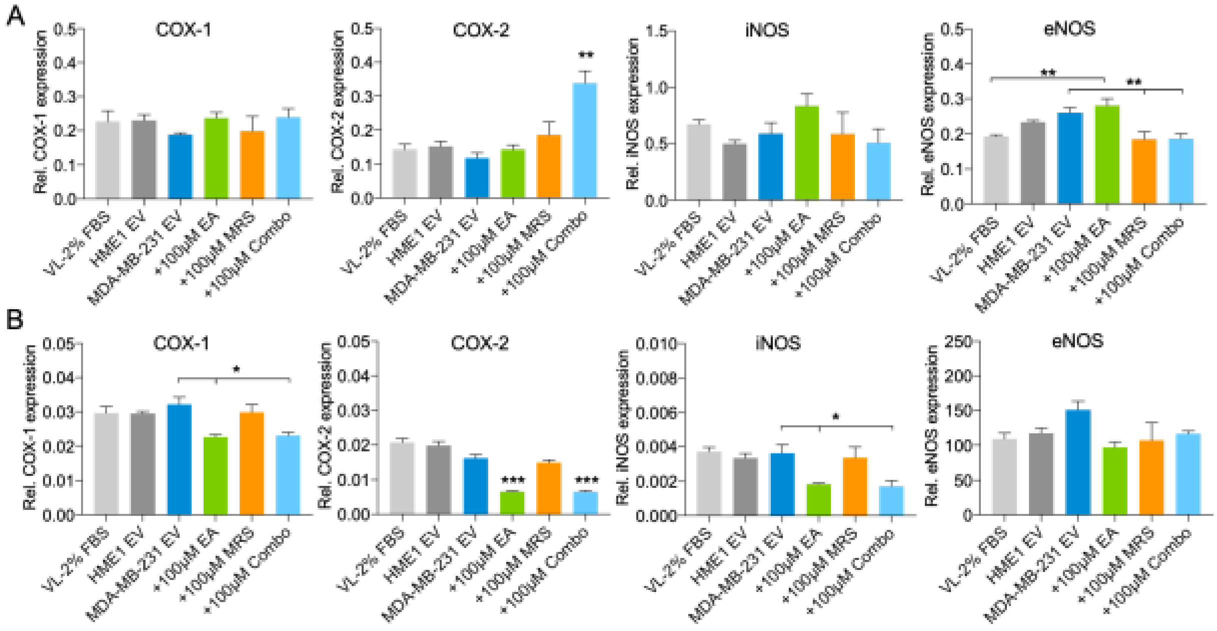

Supplementary Figure S4. Expression of COX-1, COX-2, iNOS, and eNOS in three- and 24-hour treated HUVECs. *(A)* Wes analysis of three-hour treated HUVECs with EVs (100 ng/ml) and high dose drug inhibitors (100 μM EA, MRS2179, or both). *(B)* Western blot analysis of 24-hour treated HUVECs with EVs (100 ng/ml) and a lower dose of drug inhibitors (10 μM EA, MRS2179, or both). n = 3. Mean ± S.E.M. *$p < 0.05$, **$p < 0.01$, and ***$p < 0.001$ by one-way ANOVA and Tukey post-test.

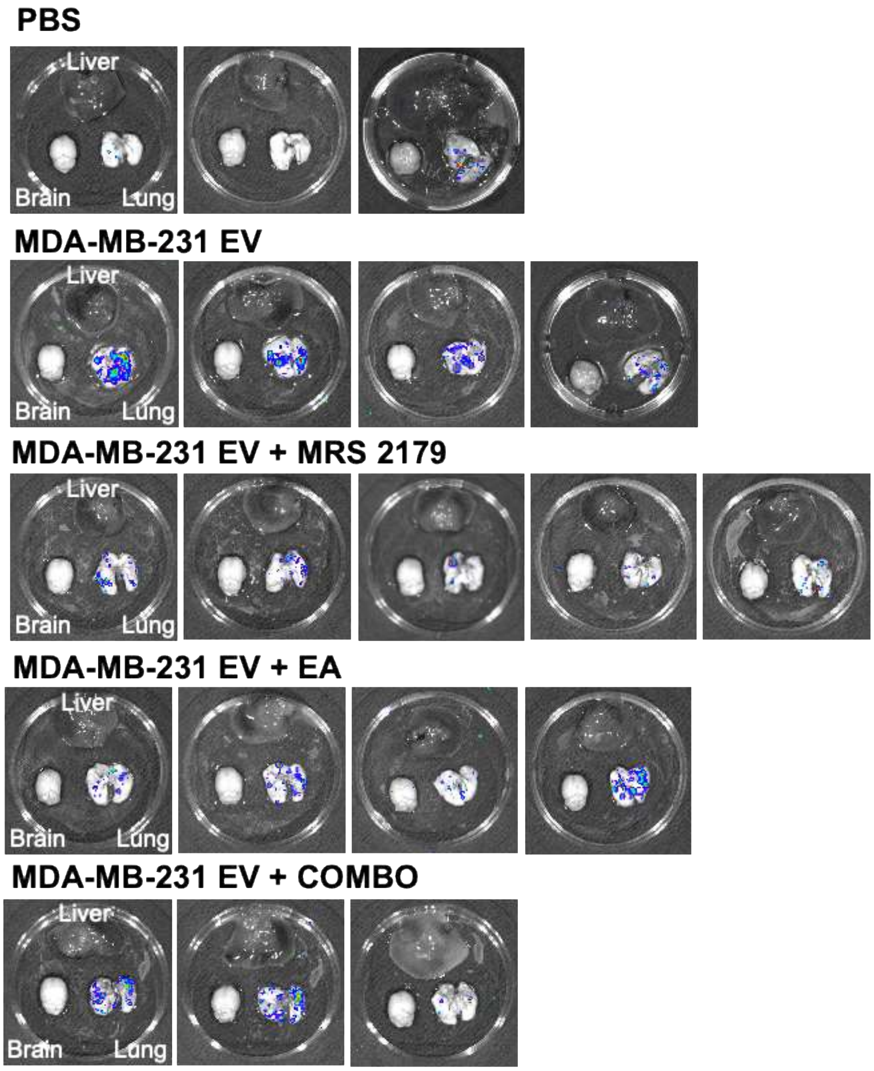

Supplementary Figure S5. Bioluminescence images of experimental metastases in brain, lung, and liver tissue from MDA-MB-231 EV treated mice. Mice were treated for eight weeks with vehicle, MDA-MB-231 EVs, or MDA-MB-231 EVs with the P2Y1 receptor antagonist MRS2179, the NDPK inhibitor ellagic acid (EA), or a combination of both drugs (Combo). Following treatment, MDA-MB-231-Luc^{+} cells were injected by tail vein and development of metastases at select organs was evaluated by bioluminescence imaging 30 days later.

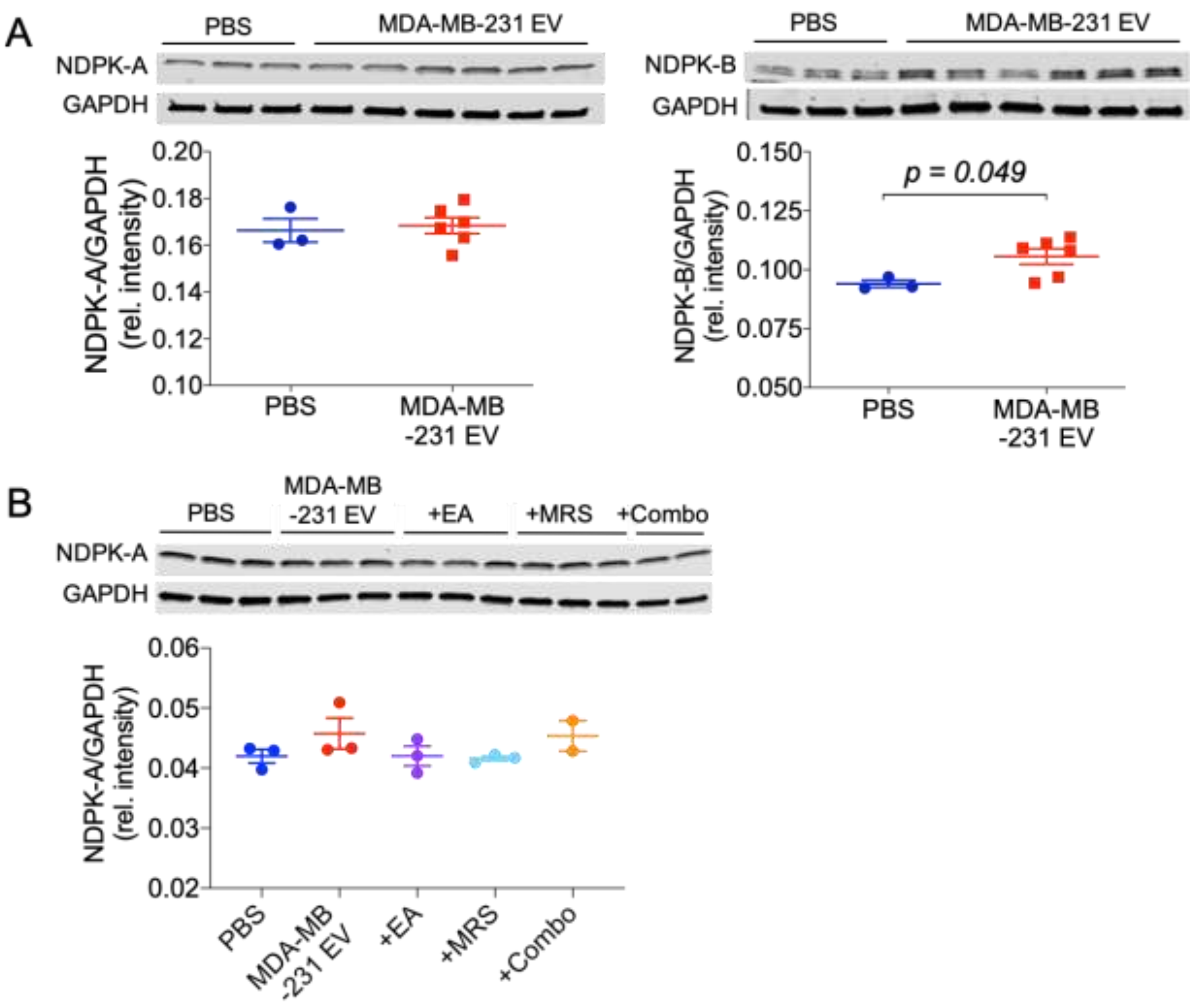

Supplementary Figure S6. Western blot analysis of NDPK-A and NDPK-B expression in MDA-MB-231 EV treated mouse lungs. *(A)* NDPK-A and NDPK-B expression in lung lysates of mice treated for three weeks with PBS (n = 3) or MDA-MB-231 EVS (n = 6). Quantitation of integrated band density shown in associated scatter plots; $p < 0.05$ by unpaired Student's T-test. *(B)* NDPK-A expression in lung lysates of mice treated for eight weeks with the indicated treatments. Following eight weeks of treatment, mice were injected by tail vein with MDA-MB-231-Luc$^+$ cells and lungs were evaluated 30 days following injection (n = 3).

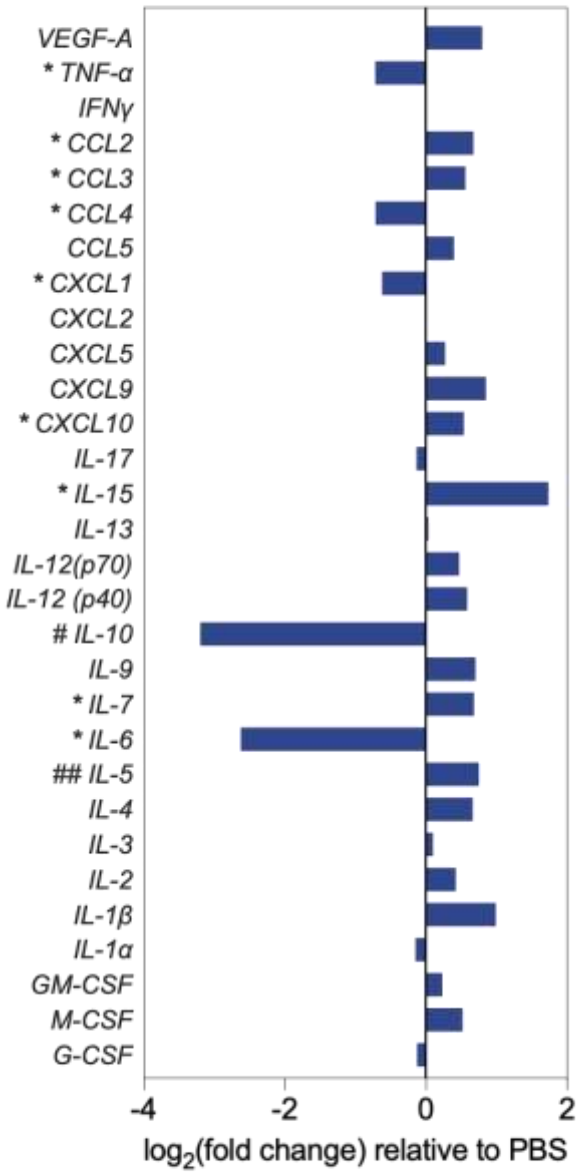

Supplementary Figure S7. Prolonged MDA-MB-231 EV exposure induces opposing actions on systemic cytokine production. Circulating cytokines were evaluated in the sera of SCID mice that were treated for eight weeks with MDA-MB-231 EVs or PBS vehicle. n = 4 per group; * = $p < 0.05$, # = $p = 0.05$, ## = $p = 0.06$ by unpaired Student's t-test. Values reflect means of $\log_2$ (fold change) relative to PBS control group.

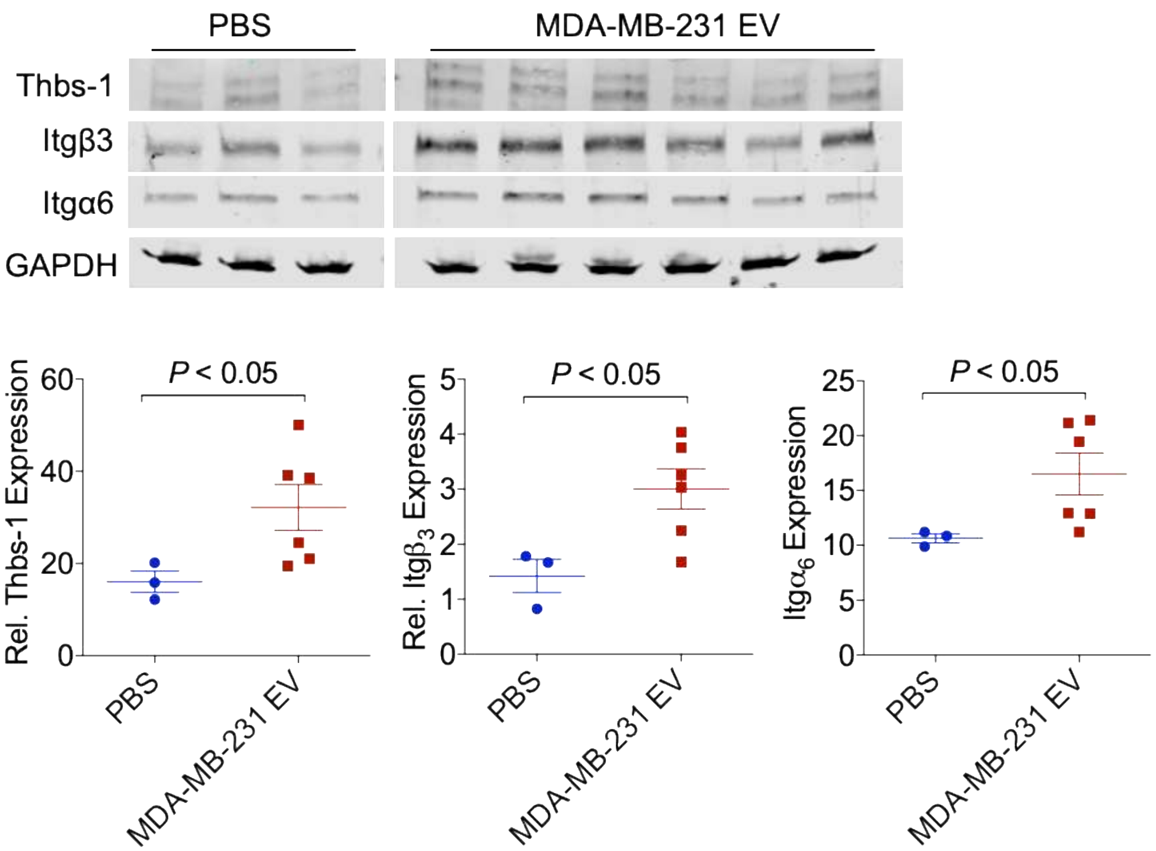

Supplementary Figure S8. Validation of mass spectrometry analysis by western blot of select differentially upregulated proteins in lungs of MDA-MB-231 EV-treated mice. NCr/SCID mice were injected by tail vein three times a week for three weeks with PBS vehicle or MDA-MB-231 EVs (10 μg). Lung lysates were analyzed by western blot for the expression of differentially expressed proteins, as previously identified by mass spectrometry. n = 3, PBS treated. n = 6, MDA-MB-231 EV treated. Mean ± S.E.M. *$P < 0.05$ by two-tailed Mann-Whitney test.

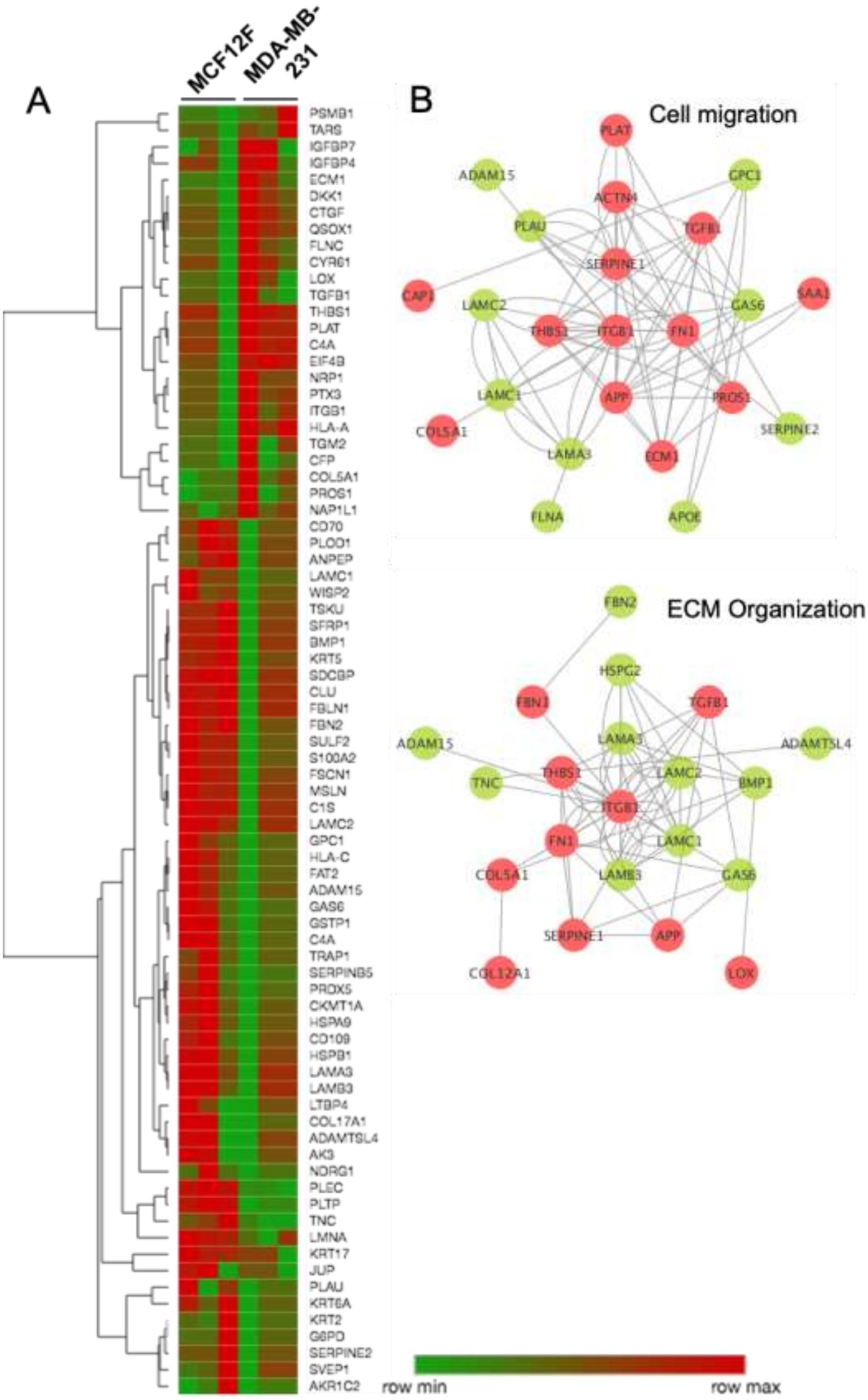

A
MCF12F
MDA-MB-231
PSMB1
TARS
IGFBP7
IGFBP4
ECM1
DKK1
CTGF
QSOX1
FLNC
CYR61
LOX
TGFB1
THBS1
PLAT
C4A
EIF4B
NRP1
PTX3
ITGB1
HLA-A
TGM2
CFP
COL5A1
PROS1
NAP1L1
CD70
PLOD1
ANPEP
LAMC1
WISP2
TSKU
SFRP1
BMP1
KRT5
SDCBP
CLU
FBLN1
FBN2
SULF2
S100A2
FSCN1
MSLN
C1S
LAMC2
GPC1
HLA-C
FAT2
ADAM15
GAS6
GSTP1
C4A
TRAP1
SERPINB5
PRDX5
CKMT1A
HSPA9
CD109
HSPB1
LAMA3
LAMB3
LTBP4
COL17A1
ADAMTSL4
AK3
NDRG1
PLEC
PLTP
TNC
LMNA
KRT17
JUP
PLAU
KRT6A
KRT2
G6PD
SERPINE2
SVEP1
AKR1C2
B
Cell migration
ECM Organization
row min
row max

Supplementary Figure S9. Proteomic profiling of ExoQuick-TC-isolated MDA-MB-231 EVs reveals malignant features consistent with parent cell line. *(A)* Heatmap with hierarchical clustering of significant differentially expressed (DE) proteins with over three-fold log2 expression difference between MDA-MB-231 EVs and MCF-12F EVs. n = 3 per group. *(B)* Significant DE proteins were mapped to biological processes associated with MDA-MB-231 EVs. Proteins with red nodes denote enrichment in MDA-MB-231 EVs, while green nodes represent proteins significantly upregulated in MCF-12F EVs.

Supplemental Materials

Expanded Methodology

Super-Resolution Microscopy. For Supplemental Figure 2, EVs were fixed onto fibronectin-coated glass slides in 4% PFA, then blocked in 5% BSA and incubated in 1:100 CPTC-NME2-3 primary antibody overnight (Developmental Studies Hybridoma Bank, Iowa City, IA). Slides were incubated in 1:100 AlexaFluor 680 secondary antibody (Thermo Fisher Scientific) and mounted onto depression slips. Slides were imaged on the Leica GSD super resolution microscope (Leica Microsystems Inc., Buffalo Grove, IL).

Transmission Electron Microscopy (TEM). Briefly, EVs were diluted in 2% PFA and adsorbed onto Formvar-carbon coated nickel grids, washed with 50 mM glycine, and blocked with 0.5% bovine serum albumin (BSA). For immunolabeling shown in Supplemental Figure 1, grids were incubated with CD9, CD81, and CD63 antibodies (Biolegend) and secondary antibodies conjugated with 10 nm gold particles (Abcam, Cambridge, MA).

TMT-labeled Mass Spectrometry. Samples were processed according to Lundby et al. (2012) and mass tagged using a TMT 10-plex isobaric label kit (Thermo Fisher Scientific). Pooled TMT-labeled peptides were fractionated by basic pH reversed-phase fractionation on an Ultimate 3000 HPLC (Thermo Fisher Scientific). BPRP fractions were separated using an UltiMate 3000 RSLCnano system (Thermo Fisher Scientific). Mass spectral analysis was performed using an Orbitrap Fusion mass spectrometer (Thermo Fisher Scientific). TMT analysis was performed using an MS3 multi-notch approach. Data was analyzed using Sequest (Thermo Fisher Scientific, version v.27, rev. 11.) and Proteome Discoverer (Thermo Fisher Scientific, Version 2.1). A minimum coverage of two peptides was required for positive protein identification.

Protein Level Data Analysis: 4,992 proteins with abundance values in all control samples and at least three experimental samples met our criteria for further study. The abundance data of these 4,992 proteins were normalized at the protein level, following the TMT Thermo-Fisher protocol and manual (Thermo Proteome Discoverer User Guide, Version 2.1), using the sum of the abundances of a pool of the nine samples included on the multiplex. Normalized data were log2-transformed to follow a normal distribution. Control and experimental abundance values were examined by histogram and quantile (Q-Q) plot to confirm a normal distribution. As each protein was examined as an independent entity and each protein's cohorts included at least three values, the Shapiro-Wilk test was used to test for normality. 208 proteins did not pass the test for normality and were excluded from analyses. Student's t-tests were performed on the normalized data of 4,784 proteins and a correction for the false discovery rate (FDR) was applied.

Pathway Analysis: iPathwayGuide (iPG) version 1711 (Advaita Corporation, Plymouth, MI), Ingenuity Pathway Analysis (IPA) (Qiagen, Redwood City, CA), and STRING database version 10.5 (STRING Consortium) were used to identify affected pathways. A cutoff criteria of $p < 0.05$ with log2 fold-change ≥ 0.5 for differentially expressed proteins was applied. Proteins with known symbols (UNIPROT) and their corresponding expression values were uploaded into each database and protein symbols were mapped to their corresponding genes. For IPA, canonical pathways most significant to the input data set were identified using the IPA library of canonical pathways. Significance was based on two parameters: (1) A ratio of the number of genes from the data set that map to the pathway divided by the total number of genes that map to the canonical pathway and (2) a P value calculated using Fisher's exact test determining the probability that the association between the genes in the data set and the canonical pathway is due to chance alone. The log2 normalized expression values of the differentially expressed proteins were mapped using the open source online-tool Morpheus (Broad Institute).

Experimental Metastasis Study: For cytokine analysis presented in Supplemental Figure 7, serum from eight-week PBS or MDA-MB-231 EV treated mice was analyzed using CD32 and Angio 5-plex cytokine arrays (Eve Technologies, Calgary, Canada).

Catalogue of Primary Antibodies and Dilutions:

Primary Antibodies	Cat #	Conc. mg/ml	Dilution	Clone	Source
CD9	312102	0.5	1:1000	HI9a	Biolegend
CD63	cbl553	0.1	1:250	RFAC4	Millipore
CD63	353013	1	1:1000	H5C6	Biolegend
CD81	348501	0.5	1:1000	5A6	Biolegend
ALIX	634501	0.5	1:1000	3A9	Biolegend
Flotillin-1	849801	0.5	1:1000	W16108A	Biolegend
Calnexin	699401	0.5	1:1000	W17077	Biolegend
Tsg101	sc-136111	0.1	1:250	51	Santa Cruz
MFGE8	sc-271574	0.2	1:500	F-5	Santa Cruz
PE-mouse IgG1 k isotype	400113	0.2	1:100	MOPC-21	Biolegend
PE anti-human CD63	353003	0.1	1:100	H5C6	Biolegend
PE anti-human NDPK-A	N7100-66A	0.1	1:100	-	US Biologicals
Rab anti-human NM23A	Ab92327	0.1	1:200	EPR3036	Abcam
Rab anti-human NME2	Ab131329	0.1	1:200	EPR8351	Abcam
PE donkey anti-Rabbit	406421	0.2	1:200	Poly4064	Biolegend
Rabbit IgG isotype	Ab172730	0.1	1:200	EPR25A	Abcam
CPTC-NME1-5	-	0.03	1:1000	-	DSHB
CPTC-NME2-1	-	0.47	1:1000	-	DSHB
Firefly Luciferase	Ab21176	11.7	1:1000	-	Abcam
Thrombospondin-1	Ab85762	1	1:1000	-	Abcam
GAPDH	D16H11	-	1:1000	5174	CST
COX-1	4841S	-	1:1000	9896	CST
COX-2	4842S	-	1:1000	12282	CST
NOS2 (iNOS)	SC-650	0.2	1:1000	M-19	Santa Cruz
NOS3 (eNOS)	SC654	0.2	1:1000	C-20	Santa Cruz
ICAM-1 (CD54)	116102	0.5	1:250	YN1	Biolegend
ICAM-2 (CD102)	105601	0.5	1:250	3C4	Biolegend
VCAM-1 (CD106)	105702	0.5	1:250	429	Biolegend
ZO-1	D7D12	-	1:250	8193	CST
β-catenin	D10A8	-	1:250	8480	CST
Integrin β3	SC-6627	0.2	1:1000	N-20	CST
GAPDH	SC-47724	0.2	1:1000	0411	Santa Cruz
